# Nanotechnology for the Poor?

**DOI:** 10.1371/journal.pmed.0020280

**Published:** 2005-08-30

**Authors:** Guillermo Foladori, Noela Invernizzi

**Affiliations:** **1**Universidad Autonoma de ZacatecasZacatecas, Mexico

After interviewing 63 experts, Salamanca-Buentello et al. [1] identified the ten main nanotechnologies that could provide a solution to problems involving water, agriculture, and health. Overflowing with good intentions, the proposal reflects the idea that if a problem can be identified, all that has to be done is apply a suitable technology and it will be solved. Most of the examples do not take into account that the relationship between science and society is much more complex.

The authors suggest that quantum dots could detect HIV molecules in the early stages, facilitating the treatment and reducing the number of new cases. The authors seem to forget the story of recent years, which has been one of open war between multinational pharmaceutical corporations and countries seeking to manufacture antiretrovirals. Nanotechnology products are already being patented. A patent in the US costs US$30 000 in legal bureaucracy, and a worldwide patent may be as much as US$250 000 [2]. The moral of the story: the efficiency and implications of the application of technology depend on the social context.

The article identifies nanotechnology as the solution to five of the eight UN Millennium Development Goals [3]. Among these solutions are nanosensors to improve the dosage of water and fertilization of plants, and hopefully reduce poverty and hunger. Not so long ago, genetically modified organisms were hailed as the solution that would put an end to hunger. However, they ended up being used mainly in developed countries. There has been no improvement for developing countries; quite the contrary, transgenics turned up where they were not wanted, as was the case in Oaxaca [4]. The moral of the story: the choice of technology is not a neutral process. It is not necessarily true that the technology that is best and meets our needs will be the one to survive.

In a previous article [5] three of the same authors maintained that the position adopted by Prince Charles—arguing that nanotechnology will widen the gap between rich and poor countries—and the position of the Action Group on Erosion, Technology and Concentration—requesting a moratorium on manufacture and commercialization of synthetic nanoparticles—both ignore the voices of people in developing countries. With their research the authors intended to fill this gap. But the opinion of scientists involved in nanotechnology does not necessarily fall in with the most appropriate pathways for satisfying the needs of the poor. We may concur that infectious diseases are one of the main problems that the developing world is facing, but we may differ radically on how a solution to this problem should be attained. Prevention is not the same thing as cure. Nanotechnology is not necessary to reduce malaria radically, as is suggested by the authors. In Henan Province, China, malaria was reduced by 99% between 1965 and 1990 as a result of social mobilization, backed up by fumigation, the use of mosquito nets, and traditional medicine based on artemisinin [6]. Viet Nam reduced the number of malaria-related deaths by 97% between 1992 and 1997 with similar methods [7]. The moral of the story: there are many means to an end, and technology is not always the solution. Organizing people can be just as important.
